# Acupuncture for chronic neck pain: a protocol for an updated systematic review

**DOI:** 10.1186/s13643-016-0257-x

**Published:** 2016-05-04

**Authors:** Qinhong Zhang, Jinhuan Yue, Xiangxin Zeng, Zhongren Sun, Brenda Golianu

**Affiliations:** Department of Anesthesiology, Stanford University, 300 Pasteur Dr, Stanford, CA 94305 USA; Department of Acupuncture and Moxibustion, College of Acupuncture and Moxibustion, Heilongjiang University of Chinese Medicine, Harbin, 150040 China

**Keywords:** Chronic neck pain, Acupuncture, Systematic review, Randomised controlled trial

## Abstract

**Background:**

This study aims to investigate the efficacy and safety of acupuncture for patients with chronic neck pain.

**Methods:**

The MEDLINE, EMBASE, CENTRAL, CINAHL, and the Chinese Biomedical Literature Database, the China National Knowledge Infrastructure, VIP Information, and Wanfang Data databases will be searched from their inception to present. Randomised controlled trials (RCTs) of acupuncture (assessed as the sole treatment or as an adjunct treatment) for chronic neck pain will be included. The primary outcome is chronic neck pain measured by the visual analogue scale (VAS), McGill Pain Questionnaire, or short-form McGill Pain Questionnaire. The secondary outcomes will include the functional recovery, health-related quality of life, psychological improvements related to the reduction of pain, and adverse events. Two authors will perform the study selection, data extraction, and quality assessment independently. Any disagreements will be resolved through discussion with a third author. Methodological quality of the included trials will be evaluated by the Cochrane risk-of-bias criteria, and the Standards for Reporting Interventions in Controlled Trials of Acupuncture checklist will be used to assess completeness of reporting.

**Discussion:**

The results of this systematic review will provide the latest evidence of the efficacy of acupuncture in treating chronic neck pain, which will benefit both practitioners and policymakers.

**Systematic review registration:**

PROSPERO CRD42015017178

**Electronic supplementary material:**

The online version of this article (doi:10.1186/s13643-016-0257-x) contains supplementary material, which is available to authorized users.

## Introduction

Neck pain is a common medical condition and a common cause of disability [1, 2]. It is reported that a 12-month prevalence of neck pain is 30–50 % in the adult population [3]. Additionally, 23 % of individuals will develop a recurrent episode in the months after their recovery [4], and women are more likely than men to develop neck pain [4].

Multiple physical interventions are available to treat chronic neck pain, such as exercise [5, 6], traction [7, 8], physical therapy [9], manual therapy [10, 11], massage, and others [12]. However, a previous study, based on a rigorous assessment of randomised controlled trials (RCTs), found no clear evidence that any type of physical therapy was more efficient than any other for chronic neck pain [13].

Acupuncture, a physical intervention which involves placement of small needles in the skin at different acupoints, has been practiced in China for 2000 years and is commonly used for many types of chronic pain [14–18]. It is believed that acupuncture relieves pain by preventing or modifying peripheral, spinal, and supraspinal mechanisms [19]. The efficacy of acupuncture for neck pain has been evaluated in three systematic reviews [18, 20, 21]. Although previous systematic reviews of neck pain have provided some insight into the potential benefit of acupuncture, this study aims to update the previous systematic review and to further specifically and critically evaluate the clinical efficacy and safety of acupuncture for chronic neck pain.

### Objectives

We will conduct a systematic review to critically assess the efficacy and safety of recent clinical evidence of acupuncture for chronic neck pain.

## Methods

### Study registration

The protocol for this systematic review has been registered with PROSPERO 2015 (http://www.crd.york.ac.uk/PROSPERO) under registration number CRD42015017178. This protocol is performed and reported according to the Preferred Reporting Items for Systematic Reviews and Meta-Analyses Protocols (PRISMA-P) statement guidelines (see Additional file 1) [22]. However, the review will be conducted depending on the Preferred Reporting Items for Systematic Reviews and Meta-Analyses (PRISMA) statement guidelines [23].

### Criteria for study inclusion

#### Study types

RCTs which compare acupuncture with sham acupuncture or other interventions in patients with chronic neck pain will be included. Non-randomised studies will be excluded. No language restriction will be applied in this study.

#### Participants

Studies evaluating patients diagnosed with chronic neck pain for at least 3 months will be included regardless of their age, sex, or ethnicity.

In addition, studies of chronic neck pain related to the following diagnostic categories will be included: mechanical neck disorders, including whiplash-associated disorders categories 1 and 2 [24, 25], myofascial chronic neck pain, and degenerative changes [26] and neck disorder with radicular symptoms, including whiplash-associated disorder category III, but without headache [24, 25]. Furthermore, if the participants have other types of pain in addition to chronic neck pain, we will only focus on the chronic neck pain. However, studies of chronic neck pain related to fractures and dislocations, coexisting headache or headache; other pathological entities; and definite or possible long tract signs (e.g. myelopathies) will be excluded.

#### Interventions

Acupuncture therapy involving body, scalp, auricular acupuncture, and electroacupuncture will be included. However, studies comparing different types of acupuncture or different points will be excluded.

Comparison interventions may be sham acupuncture (including minimal acupuncture, using of invalid points, using invalid stimulation but on appropriate location or depth, and non-penetrating sham device such as Park’s sham device or mock electrical stimulation) or other therapies including no treatment, usual care, and other conventional treatments. In addition, we will include studies assessing acupuncture combined with another non-acupuncture intervention compared with the non-acupuncture intervention alone.

#### Outcome measures

##### Primary outcomes

Chronic neck pain will be assessed by the visual analogue scale (VAS) (0–100) [27], McGill Pain Questionnaire (MPQ), or short-form McGill Pain Questionnaire (SF-MPQ) [28–30].

##### Secondary outcomes

Secondary outcomes will include functional recovery (such as disability, return to activities, work, or school), health-related quality of life [31], and psychological improvements related to the reduction of pain. Additionally, side effects related to the acupuncture intervention will also be documented.

#### Search methods to identify studies

The search strategy is developed with the help of experienced librarians to retrieve MEDLINE, EMBASE, CENTRAL, CINAHL, the Chinese Biomedical Literature Database (CBM), the China National Knowledge Infrastructure (CNKI), VIP Information (VIP), and Wanfang Data (WANFANG) for key terms from their inception to present. We will use the following search terms: pain, neck pain, acupuncture, acupuncture therapy, manual acupuncture, electroacupuncture, scalp acupuncture, controlled trial, and randomised controlled trial. Chinese translation of the same search terms will be used in the Chinese databases. The search strategy for CENTRAL is shown in Table 1. In addition, the reference lists of previously published reviews related to acupuncture and chronic neck pain will be searched.

**Table 1:** Search strategy used in CENTRAL database

Number	Search terms
1	MeSH descriptor: [chronic neck pain] explode all trees
2	((neck muscles) or (cervical plexus) or (cervical vertebrae) or (Atlanto-Axial Joint) or (atlanto-occipital joint) or (spinal nerve roots) or (brachial plexus)):ti, ab, kw
3	or 1–2
4	MeSH descriptor: [acupuncture] explode all trees
5	MeSH descriptor: [acupuncture therapy] explode all trees
6	((manual acupuncture) or (manual next acupuncture*) or (electroacupuncture) or (electro next acupuncture*) or (scalp acupuncture*) or (scalp next acupuncture*)):ti, ab, kw
7	or 4–6
8	MeSH descriptor: [randomized controlled trial] explode all trees
9	((random) or (clinical trial*) or (clinical next trial*) or (controlled clinical trial) or (controlled next clinical trial*)):ti, ab, kw
10	or 8–9
11	3 and 7 and 10

This search strategy will be modified as required for other electronic databases

### Data collection and analysis

#### Study selection

**Fig. 1:**
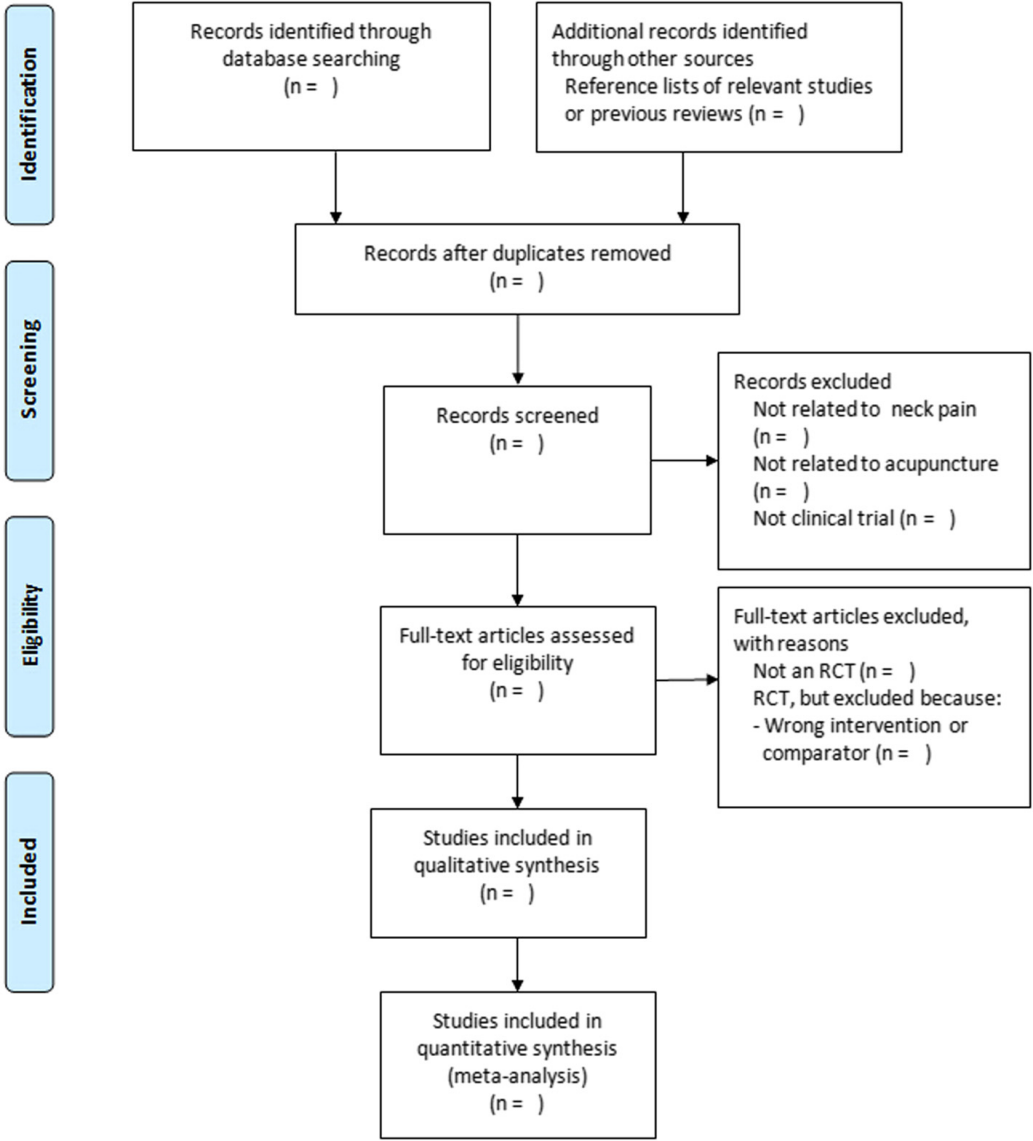
Flow diagram of the trial selection process

All studies will be screened based on their titles and abstracts first by two review authors (X.X.Z. and J.H.Y.) independently. After that, the full text will be reviewed and eligible studies will be selected. Potential disagreements will be resolved by discussion with a third review author (Q.H.Z.). The whole process of study selection is summarised in the PRISMA flow diagram (Fig. 1).

#### Data extraction

Data are extracted from included trials by two authors (J.H.Y. and Q.H.Z.) independently according to a predefined data extraction sheet. The extracted data will include the author, title, publication year, journal, location, participants, study size, randomisation, allocation concealment, blinding, interventions (acupuncture and control group), main outcomes, duration, follow-up, adverse events, withdrawals, and conflicts of interest. Reporting will be assessed for completion by utilising the Standards for Reporting Interventions in Controlled Trials of Acupuncture (STRICTA) checklist [32].

#### Quality assessment

##### Assessment of risk of bias in included studies

The Cochrane risk of bias tool [33] and the STRICTA checklist for reporting intervention details of acupuncture will be used to evaluate the risk of bias and completeness of reporting of acupuncture intervention, respectively. All evaluations will be performed by two independent reviewers (Y.J.H. and Z.Q.H.). All discrepancies will be resolved by discussion with a third author (G. B. or S.Z.R.).

##### Measures of treatment effect

For continuous outcomes, such as VAS or MPQ, the mean difference (MD) with a 95 % confidence intervals (CIs) will be used. For dichotomous data (e.g. adverse events), risk ratio (RR) with 95 % CIs will be used. Other forms of continuous or dichotomous data will be converted into MD or RR values, respectively.

#### Missing data

We will attempt to acquire any missing data by contacting the original study authors whenever possible. If it is not possible to get the missing data, then only the available data will be analysed.

#### Assessment of heterogeneity

We will evaluate heterogeneity according to the description in Section 9 of the Cochrane Handbook [34]. If significant heterogeneity exists, we will perform a subgroup analysis to explore the possible causes [34].

#### Assessment of reporting biases

We will use funnel plots to detect potential reporting biases. It will be used to analyse the asymmetry if at least ten trials are available in the meta-analysis [35].

#### Data synthesis

If it is possible to conduct a meta-analysis, Review Manager (version 5.3) software [36] (the Cochrane Collaboration, Oxford, England) will be used to combine the RR for dichotomous outcomes and MD for continuous data both with 95 % CIs. Random effects model will be used if high level of clinical heterogeneity is expected due to a diverse style of acupuncture practice, different non-clinical backgrounds (country, culture, or healthcare systems) and trial settings. Otherwise, we will apply a fixed effects model. If any meta-analysis cannot be performed, we will report the results as the narrative description.

#### Subgroup analysis

Subgroup analysis will be conducted to assess the heterogeneity between the included trials. The analysis will include the type of acupuncture and control, such as acupuncture versus sham acupuncture (including non-penetrating acupuncture) and acupuncture versus active comparator.

#### Sensitivity analysis

Sensitivity analysis will be conducted by excluding the included studies at high risk of bias for any one or more of selection, attrition, or detection bias. The meta-analysis will be repeated after removing the lower quality trials. The results will be compared and discussed according to the pooled effect size.

## Discussion

Acupuncture utilisation continues to increase, with over 3 million adults undergoing acupuncture every year, often for chronic pain [37, 38]. Initial analyses suggest that acupuncture may also be a cost-effective intervention in the management of a number of painful conditions, including headache, neck pain, and back pain [39–41]. The evidence base to guide its rational use needs to be continually updated and revised to update standards of care.

Three previous systematic reviews have analysed randomised controlled trial acupuncture treatment of neck pain [18, 20, 21]. While some studies have excellent methodology, the heterogeneity of the study protocols prevented them from being able to draw firm conclusions about the efficacy of acupuncture. Thus, a new updated, comprehensive, and objective systematic for the clinical efficacy and safety of acupuncture for chronic neck pain would be of benefit to provide the needed evidence base for further treatment recommendations. This systematic review will provide a detailed summary and latest analysis of the current evidence for the efficacy of acupuncture in treating chronic neck pain, which will inform patient care, as well as health policy.

### Stage of review at PROSPERO

The stage of review at PROSPERO is data extraction.

## Additional files

Additional file 1: PRISMA-P checklist. PRISMA-P (Preferred Reporting Items for Systematic review and Meta-Analysis Protocols) 2015 checklist: recommended items to address in a systematic review protocol. (DOC 90 kb)

## Abbreviations

CBM: Chinese Biomedical Literature Database
CIs: confidence intervals
CNKI: China National Knowledge Infrastructure Database
MD: mean difference
MPQ: McGill Pain Questionnaire
PRISMA: Preferred Reporting Items for Systematic Reviews and Meta-Analyses
PRISMA-P: Preferred Reporting Items for Systematic Reviews and Meta-Analyses Protocol
RCTs: randomised controlled trials
RR: risk ratio
SF-MPQ: short-form McGill Pain Questionnaire
STRICTA: Standards for Reporting Interventions in Controlled Trials of Acupuncture
VAS: visual analogue scale
VIP: VIP Information
WANFANG: Wanfang Data
